# How does AI perform compared to human expert panels in medical Delphi studies? A pilot study through the lens of pathology

**DOI:** 10.1016/j.jpi.2026.100661

**Published:** 2026-04-15

**Authors:** Joshua Pantanowitz, Christopher D. Manko, John Majewski, M. Alvaro Berbís, Alton B. Farris, Aly Karsan, Andrey Bychkov, Antonio Luna, Bethany Williams, Brett Delahunt, Catarina Eloy, David S. McClintock, Eva Hollemans, J. Mark Tuthill, Jeroen Van der Laak, Jerome Y. Cheng, Jochen K. Lennerz, John H. Sinard, Jose Aneiros-Fernandez, Julien Calderaro, Kenneth A. Iczkowski, Lars Egevad, Mark Kriegsmann, Markus D. Herrmann, Mohamed E. Salama, Raimundo Garcia Del Moral, S. Joseph Sirintrapun, Colleen Vrbin, Liron Pantanowitz, Jeanne Shen, Hooman Rashidi

**Affiliations:** a University of Pittsburgh School of Medicine, Pittsburgh, PA, USA; b Geisinger Commonwealth School of Medicine, Scranton, PA, USA; c Department of Internal Medicine, Wexner Medical Center, The Ohio State University College of Medicine, Columbus, OH, USA; d Department of R&D, HT Médica, San Juan de Dios Hospital, Córdoba, Spain; e Department of Pathology and Laboratory Medicine, Emory University, Atlanta, GA, USA; f Department of Pathology & Laboratory Medicine, University of British Columbia, BC Cancer Research Institute, Vancouver, Canada; g Department of Pathology, Kameda Medical Center, Kamogawa, Chiba, Japan; h Department of Integrated Diagnostics, HT Médica, Clínica Las Nieves, Jaén, Spain; i Department of Histopathology, Leeds Teaching Hospitals NHS Trust, Leeds, UK; j Malaghan Institute of Medical Research, Wellington, New Zealand; k Department of Pathology - Oncology, Karolinska Institute, Stockholm, Sweden; l Pathology Laboratory, Institute of Molecular Pathology and Immunology, University of Porto, Porto, Portugal; m Department of Laboratory Medicine and Pathology, Mayo Clinic, Rochester, MN, USA; n Department of Pathology, Erasmus University Medical Center, Rotterdam, the Netherlands; o Pathology Informatics, Department of Pathology, Henry Ford Hospital, Detroit, MI, USA; p Department of Pathology, Radboud University Medical Center, Nijmegen, the Netherlands; q Department of Pathology, University of Michigan, Ann Arbor, MI, USA; r BostonGene, Waltham, MA, USA; s Department of Pathology, Yale University School of Medicine, New Haven, CT, USA; t Department of R&D, HT Médica, San Juan de Dios Hospital, Córdoba, Spain; u Department of Pathology, Henri Mondor-Albert Chenevier University Hospital, Assistance Publique-Hôpitaux de Paris, Créteil, France; v Department of Pathology and Laboratory Medicine, University of California Davis Health, Sacramento, CA, USA; w Department of Oncology-Pathology, Karolinska Institute, Stockholm, Sweden; x Center for Histology, Cytology and Molecular Pathology Wiesbaden, Wiesbaden, Germany; y F. Hoffmann-La Roche Ltd, Basel, Switzerland; z Department of Pathology, Sonic Healthcare, Austin, TX, USA; aa Department of Pathological Anatomy and History of Science, University of Granada, and Institute of Biosanitary Research of Granada (ibs.GRANADA), Granada, Spain; ab Department of Pathology, Mass General Brigham, Harvard Medical School, Boston, MA, USA; ac Analytical Insights LLC, Allison Park, PA, USA; ad Computational Pathology and AI Center of Excellence, Department of Pathology, University of Pittsburgh Medical Center and School of Medicine, Pittsburgh, PA, USA; ae Department of Pathology and Center for Artificial Intelligence in Medicine & Imaging, Stanford University School of Medicine, Stanford, CA, USA

**Keywords:** Delphi study, Artificial intelligence, Large language model, Pathology, Medicine, Temperature setting

## Abstract

**Background:**

Since their inception, Delphi studies have been a key part of medical literature. They consist of an expert panel tasked with coming to consensus on answers to various questions where obtaining objective results is difficult or impossible, with ranked responses based on a Likert scale. The ability of artificial intelligence (AI), particularly large language models (LLMs), to perform this role traditionally assigned to a panel of experts has been scarcely explored in medicine. This study accordingly aimed to explore the feasibility of an “AI-run” Delphi study applied to the practice of pathology.

**Methods:**

A prior human-based Delphi study (PMID: 36603288) employed to forecast the future role of AI in pathology was repeated, but this time with LLMs (Llama 3, ChatGPT-4, and ChatGPT-3.5 based on availability at the time of the study). This was done at various temperature settings (0, 0.7, and 1.0), a measurement of how much an LLM prioritizes determinism versus creativity. Low temperature caused the models to be more deterministic and focused, whereas high temperature increased creativity. “Delphi-GPT” was created to automate prompts that entailed 5 trials for 180 questions, leading to data that were compared to the original human expert panel.

**Findings:**

All LLM and temperature combinations were able to reach consensus for a greater percentage of the 180 questions posed than human experts. Newer ChatGPT-4 and Llama 3 models performed better than ChatGPT-3.5. Whereas AI models and human experts did not always agree, the amount of agreement increased when the temperature setting was increased across all LLMs.

**Interpretation:**

LLMs are shown here to successfully be able to simulate a Delphi study in medicine. The data show that generative AI models were consistently able to reach greater degrees of consensus than human experts in their responses to 180 prompts related to the future practice of pathology. This serves as a proof-of-concept that one day, pending further robust methodological validation, AI could even serve as a surrogate for de novo Delphi studies that ordinarily would have relied on feedback from a panel of experts. The reliability of consensus/concordance achieved will depend upon the combination of LLM and temperature setting selected.

## Introduction

The origin of the Delphi technique has been attributed to the United States military in the 1950s as a defense strategy, in order to achieve a consensus on the opinion of experts while avoiding problems that come with more confrontational survey methods such as round-table discussions, which can lead to dominance by group members with stronger personalities and groupthink.^2^, ^3^

The Delphi technique works by initially asking a panel of anonymous human experts for their opinion on a particular problem. The dataset formed from the experts' opinions is then analyzed and helps form the questions then posed to those experts in each subsequent round. Often the questions are presented as a survey using Likert scale rankings. Participants also see the anonymous results of each previous round to consider feedback and opinions of other experts in a non-confrontational manner. These rounds continue, with each round of questions formed from previous findings, until a consensus is reached.^4^

One major flaw with such human-based Delphi studies is that there is no agreed-upon definition of what “consensus” is, and it is unlikely to achieve full agreement on all presented issues. Thus, it is important to have a predetermined consensus level before beginning a Delphi study.^5^ A previous systematic review of Delphi studies found that most of the Delphi studies observed did not have formal criterion for consensus, yet consensus was determined in the majority of studies.^6^ Human consensus studies are also subject to poor organization, bullying and failed conclusions.

The Delphi technique has expanded beyond its original use in the military and has been widely applied to the health sciences. It is most useful in the health sciences to collect a consensus opinion in an area, where little empirical research is available, or to help answer a healthcare question that has no definitive answers. This can help guide future research based on the consensus opinions reached by the Delphi study.^7^

Recent examples of using the Delphi method in healthcare include studies evaluating policies that would help lead to sustainable healthcare systems in Korea and Australia.^8^, ^9^ Further, many Delphi studies have been published in recent years investigating the role of artificial intelligence (AI) in numerous medical and non-medical applications.^1^, ^10^, ^11^, ^12^, ^13^, ^14^

However, a novel idea which has been hitherto scarcely explored is, given the extensive time needed to acquire iterative human expert consultation in Delphi studies, whether AI may have the potential to become the expert panel in a Delphi study itself, potentially saving the research community immense time. Generative AI models such as large language models (LLMs) are feasibly well suited for this task, as they have been trained on vast textual data that can simulate expert-like responses or synthesize opinions. Indeed, limited proof-of-concepts have been performed, albeit not yet in medicine. One study by Mueller et al. found that AI could help serve as an additional source of expertise alongside a human expert panel to strengthen the Delphi study at hand, rather than replace human experts altogether.^15^ However, when these researchers attempted to use AI to directly replicate/clone expert individuals (to simulate the potential for AI use in cases where experts end up not being able to attend iterative consultations), they were unable to do so successfully.^15^ Another article by Nobrega et al., however, did find success when using AI to conduct the Delphi method in place of a panel of well-known human experts in the fields of Economics, Entrepreneurship, Politics, Computer Science, Sociology, and Philosophy.^16^ Further work by Richard Grigonis also found success with the use of AI, specifically various competitor LLMs, in a Delphi analysis.^17^

Whereas these works reveal nuance and insight into AI's potential role in conducting Delphi studies, to the authors' best knowledge, there are no Delphi studies using AI in place of an expert human panel within the health sciences. Hence, this study was conducted to explore the role that AI could play in emulating medical human expert panels for Delphi studies. The scope of this study is narrowed to the lens of pathology, as per the authors' expertise. Specifically, the human-based Delphi study used as the baseline for this analysis is a previous Delphi study published in *eBioMedicine* about predicting the future of pathology AI and computational pathology by 2030.^1^

## Methods and materials

The original *eBioMedicine* Delphi study from 2023 surveyed 24 experts in the areas of pathology AI and computational pathology, asking a total of 180 questions, of which consensus was reached among these experts for 141 questions (78.3%).^1^ Responses to all 180 questions were ranked by these human experts on a Likert scale of 1–7, where higher scores indicated a more positive outlook on the future of AI in pathology.^1^ However, the exact structure and topic of the Likert scale prompts used for each question, as well as the qualitative meanings associated with the rankings of 1–7, differed among questions. In the present study, the authors examined the original survey and classified the 180 questions into 7 categories based on common Likert rating scale and question topic, including: (1) statement agreement (with statements predicting various future trends we may/may not see come to fruition in the field of pathology), (2) estimate of how the integration of AI in the pathology setting will impact the workforce, (3) estimate the degree of task involvement for pathologists by 2030, (4) estimate the degree of task involvement of pathology technicians by 2030, (5) estimate of the probability of AI application being used routinely in pathology labs by 2030, (6) estimate of the probability of AI being integrated into routine diagnostics by 2030, and (7) estimate of the probability that this task will become fully delegated to AI, and thus performed in a fully automated manner in pathology labs by 2030.

After identifying which questions belonged to each of the seven Likert-based question categories, the questions were carefully rephrased in statement formats, to enable easier interpretation by LLMs while also retaining their initial meanings. These were then divided into seven CSV files by Likert-based question category. Subsequently, for each of the seven Likert-based question categories, a generalized template prompt was developed, including all relevant study background information, considerations, and rules, and the appropriate Likert scale to be used, each including a placeholder (“{statement}”) to later be replaced according to the code with one of the 180 questions at a time (in its rephrased statement format). The template prompt for Category 1 is shown below, whereas the others can be found in the supplemental materials:

This is part of a study about what the future of AI in pathology will look like by 2030. The expert answers to Round 1 have been transformed into a series of statements, which will now be assessed by: (1) humans and (2) AI models like yourself. In this round, you will be asked to rate each of the specified statements provided to you from the CSV file according to the Likert scale below. You will rate each specified statement one trial at a time, one specified statement per trial (i.e., one trial at a time, with each trial regarding only one specified statement).

Please answer considering only AI input, not digital pathology in a broad sense. Also, please answer according to what you believe will happen by 2030, instead of what you would like to happen*.*

All of your responses will remain anonymous to the rest of the panel experts*.*

Please rate your AGREEMENT with the specified statement according to this Likert scale*:*

1 Very strongly disagree

2 Strongly disagree

3 Disagree

4 Neither agree nor disagree

5 Agree

6 Strongly agree

7 Very strongly agree

You may only rank based on these discrete categories (1, 2, 3, 4, 5, 6, or 7), no in-betweens are acceptable. Further, giving two or more Likert rankings to avoid definitively deciding is not allowed; when in doubt, you must provide your best single Likert ranking, and instead you may acknowledge any difficulties you had in coming to a final single Likert ranking within your follow-up rationale/explanation, but still you must provide a single discrete Likert ranking as your final answer, as these data will ultimately be entered categorically into an Excel file*.*

Please additionally provide a rationale/explanation behind why you decided to give the ranking you did*.*

Specified Statement: “{statement}”

The LLMs chosen for use in this study were OpenAI's ChatGPT-3.5, OpenAI's ChatGPT-4o (referred to simply as ChatGPT-4 or -4.0 henceforth), and Meta's Llama 3. The data were collected approximately between May and August of 2024. Additionally, three different temperature settings (0, 0.7, and 1) were used. In the field of AI, temperature refers to a parameter that controls how deterministic versus stochastic an AI model's responses are. In general, increasing temperature should allow for more nuanced and detailed responses, but it also runs the risk of allowing for more erroneous or off-target information as the AI becomes freer to stray from widely accepted fact. Temperature values of 0, 0.7, and 1.0 were selected to represent three commonly used sampling regimes in LLM inference that span the typical operating range used in many LLM applications. A temperature of 0 approximates deterministic generation, whereas intermediate values around 0.7 introduce moderate stochasticity while generally preserving coherence. A temperature of 1.0 reflects higher variability in token sampling and is often used to explore the upper range of response diversity and creativity. Evaluating Delphi-GPT across these settings allowed assessment of how increasing stochasticity influences response agreement and variability.

Each LLM was prompted five times per temperature per question, and the mode, percent of responses matching mode, mean, standard deviation, median, and interquartile range (IQR; 25th percentile, 75th percentile ranking) were reported for each question to mirror the reporting methodology of the original human-based Delphi article. Five trials were generated for each question at each temperature for the purpose of sampling the stochastic output distribution of the language model across repeated prompts. This number allowed estimation of response variability while maintaining a manageable computational and analytic burden for this proof-of-concept study. Although larger numbers of repetitions could further stabilize estimates of the response distribution in future applications, where Delphi-GPT may be deployed as a validated tool, a small number of repeated generations is generally sufficient in exploratory analyses to capture broad patterns of agreement and variability across model outputs.

Responses were collected by running Delphi-GPT 21 times, once separately for each of the seven Likert categories across the three temperature settings. Within each prompting run, all five trials of each question assigned to that Likert category were prompted simultaneously in an automated fashion. Temperature was the primary parameter used to regulate stochasticity; however, as with all LLMs, additional sampling mechanisms (e.g., nucleus sampling/top-p) and backend inference variability may introduce minor non-deterministic variation in outputs even when prompts and temperature settings are held constant. For this proof-of-concept study, these additional parameters were left at their default settings. Following each run, the Delphi program outputs all responses to a single .CSV file, which can then be imported into Excel if desired. The raw Excel files are provided in the supplemental materials for full transparency and reproducibility.

**Fig. 1:**
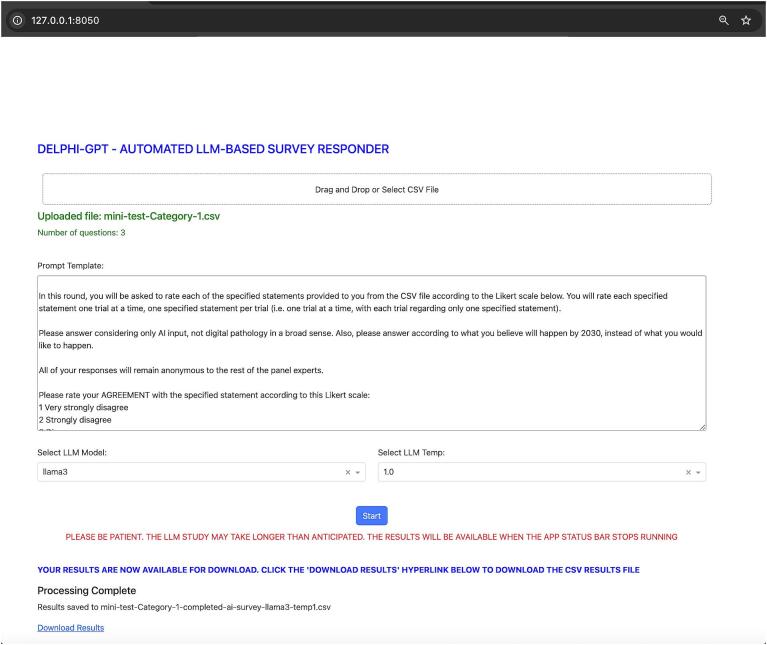
The user interface of the python-coded application, “Delphi-GPT,” used to automate prompting of the LLM models.

Specifically, Python code was developed to enable automation of this prompting methodology for the LLMs. This application was named the “Delphi-GPT.” The user interface is shown in Fig. 1. The detailed code for the application is shared in the supplemental materials. The application allows the user to input the generalized prompt template for the relevant Likert-based question category into an open-ended text box, select the CSV file to upload containing the desired questions (rephrased as statements), select which LLM model to prompt, and set the desired temperature setting. Upon running the program, it goes through each question (rephrased as a statement) in the CSV file successively, replacing the designated placeholder within the template prompt with that question's statement, and prompting the desired LLM and temperature setting with the updated prompt five times before moving onwards to the next question's statement. Finally, as mentioned above, the program collects the responses (for five trials) given by the LLM at the desired temperature, and outputs them as a new CSV data file which displays both the whole LLM response, and the isolated numerical Likert value given by the AI for each trial. The raw uploaded CSV question files and the generated CSV output data files are shared in the supplemental materials.

Each LLM provided a response to each prompt. However, the LLM response did not always follow the desired format of a number rating separated from the explanation or rationale. In these instances, the analyst reviewed the explanation or rationale to identify if a number rating was included in the text. Instances of re-coding from “invalid” to a rating provided in the explanation were recorded and explored for each of the seven categories to identify if the type of rating scale may have varied.

Concordance among reviewers was based on IQR values of ≥1, which replicates the methods used in the original Berbís et al. publication.^1^ If a rating was not provided for any of the five prompts for an LLM, or if there was a sole rating out of the five, IQR was not calculated for that question, and concordance was then considered to be unknown.

The final responses from the 24 human experts in the original Delphi study were treated as the control condition and compared to each LLM (GPT 3.5, GPT 4.0, and Llama 3) for each temperature (0, 0.7, and 1.0). For the purposes of this study, each rater was considered independent. Whereas a single LLM provided five ratings per statement per temperature, each rating was treated as an independent observation for the purposes of statistical analysis. Furthermore, the repeated LLM trials should be interpreted as independent samples from the model's output distribution rather than as independent expert opinions.

Because the ratings were based on an ordinal scale, with each question having answer options 1–7, the rank-based Mann–Whitney test with the Benjamini–Hochberg correction (using false-discovery rate of 5% to limit false positives to a rate of 1 out of 20) was used for comparisons between each LLM (5 trials per question) to the expert human panel (24 experts per question). Mann–Whitney *U*, two-tailed exact significance (α = 0.05), and corrected Benjamini–Hochberg *p*-value are reported.^18^ Analysis was performed using IBM SPSS Statistics 29 and Microsoft 365 Version 2406.

**Fig. 2:**
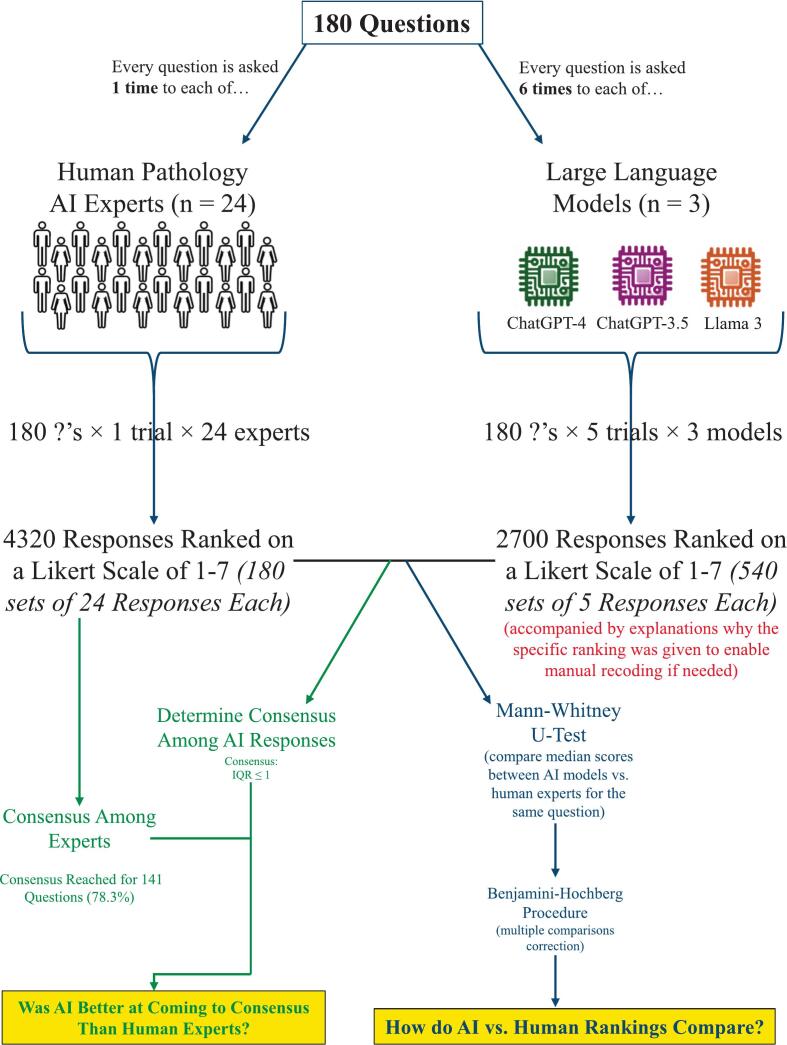
Flowchart, which illustrates the workflow of data collection and statistical analysis undertaken for the present study.

A visually summarized overview of the framework employed throughout this study is presented in Fig. 2**.**

## Results

### Recoded and excluded cases

The summary of cases per LLM and temperature that required recoding is shown in Table 1.

**Table 1:** Number of recoded responses (1+ of 5 prompts had numerical rating buried in the text explanation but not automatically extracted) by classification category.

		GPT 3.5			GPT 4.0			Llama 3			Classification category total
		Temp 0	Temp 0.7	Temp 1.0	Temp 0	Temp 0.7	Temp 1.0	Temp 0	Temp 0.7	Temp 1.0	
Classification category	1) Statement agreement	0	18	21	0	5	9	0	0	0	53
	2) Estimate of how the integration of AI in the pathology setting will impact the workforce	0	10	9	1	4	10	0	0	0	10
	3) Estimate the degree of involvement of pathologists in the task by 2030	2	12	11	0	4	11	0	0	0	15
	4) Estimate the degree of involvement of pathology technicians in the task by 2030	2	8	9	0	4	7	0	0	0	11
	5) Estimate of the probability of this application of AI being used routinely in pathology labs by 2030	38	40	41	0	23	25	0	0	0	44
	6) Estimate of the probability of this application of integrated diagnostics being used routinely by 2030	3	12	15	1	13	16	0	0	0	19
	7) Estimate of the probability that this task will become fully delegated to AI and thus done in a fully automated way in pathology labs by 2030	26	27	28	0	14	20	0	0	0	28
Total		71	127	134	2	67	98	0	0	0	–

All the Llama 3 responses were provided in the correct format (numerical rating with a separate explanation or rationale). GPT 4.0 and 3.5 both required recoding for each temperature setting. For GPT 4.0, temperature 0 had 2 cases that were recoded to include for analysis, whereas temperature 0.7 had 67 cases that were recoded, and temperature 1.0 had 98 of the 180 cases that needed recoding. The volume of recoded cases was higher for GPT 3.5, with 71 recoded cases for temperature 0, 127 recoded cases for temperature 0.7, and 134 of the 180 cases recoded for temperature 1.0.

The number of unrated responses is shown in Table 2 for each LLM and temperature. GPT 3.5 temperature 0 had the most exclusions (*n* = 30). Of the GPT 3.5 temperature 0 exclusions, all 10 of Category 2, 6 of 44 Category 5, 12 of 19 Category 6, and 2 of 28 Category 7 did not have numerical ratings provided.

**Table 2:** Number of unrated responses (5 of 5 prompts yielded text-only responses) by classification category.

		GPT 3.5			GPT 4.0			Llama 3			Classification category total
		Temp 0	Temp 0.7	Temp 1.0	Temp 0	Temp 0.7	Temp 1.0	Temp 0	Temp 0.7	Temp 1.0	
Classification category	1) Statement agreement	0	0	0	0	0	0	0	0	0	53
	2) Estimate of how the integration of AI in the pathology setting will impact the workforce	10	0	1	1	0	0	0	0	0	10
	3) Estimate the degree of involvement of pathologists in the task by 2030	0	0	0	0	0	0	0	0	0	15
	4) Estimate the degree of involvement of pathology technicians in the task by 2030	0	0	0	0	0	0	0	0	0	11
	5) Estimate of the probability of this application of AI being used routinely in pathology labs by 2030	6	0	1	0	0	0	0	0	0	44
	6) Estimate of the probability of this application of integrated diagnostics being used routinely by 2030	12	0	2	0	0	0	0	0	0	19
	7) Estimate of the probability that this task will become fully delegated to AI and thus done in a fully automated way in pathology labs by 2030	2	1	0	0	0	0	0	0	0	28
Total		30	1	4	1	0	0	0	0	0	–

Overall, using temperature 0, GPT 3.5 output did not match the required format for one or more of the 5 prompts in 101 of the 180 questions (56.1%), instead providing only a rationale without separating a rating. However, for 71 of the 101 cases, a rating was clearly provided in the rationale, and therefore, the case was recoded to include the rating resulting in a total of 30 cases (16.7%) that only provided a text rationale with no numerical rating, and therefore were unable to be used for analysis. Using temperature 0.7, GPT 3.5 output did not match the required format for one or more of the 5 prompts in 128 of the 180 questions (71.1%). Ratings for 127 of those 128 were clearly provided in the rationale, and therefore, those cases were recoded to include the rating. A total of 1 case (0.6%) only provided text rationale without a numerical rating and was therefore unable to be used for analysis. Using temperature 1.0, GPT 3.5 output did not match the required format for one or more of the 5 prompts in 138 of the 180 questions (76.7%). Ratings for 134 of the 180 were clearly provided in the rationale, and therefore those cases were recoded to include the rating. A total of 4 cases (2.2%) only provided text rationale without a numerical rating and were therefore unable to be used for analysis.

Using temperature 0, GPT 4.0 output did not match the required format for one or more of the 5 prompts in 3 of the 180 questions (1.7%), instead providing only a rationale without separating a rating. However, in two of the three cases a rating was clearly provided in the rationale, and therefore the case was recoded to include the rating. One case (0.6%) only provided a text rationale with no numerical rating, and therefore, was unable to be used for analysis. Using temperature 0.7, GPT 4.0 output did not match the required format for one or more of the 5 prompts in 67 of the 180 questions (37.2%). Ratings for all 67 of these cases were clearly provided in the rationale and therefore were recoded to include the rating. Using temperature 1.0, GPT 4.0 output did not match the required format for one or more of the 5 prompts in 98 of the 180 questions (54.4%). Ratings for all 98 of these cases were clearly provided in the rationale and therefore were recoded to include the rating.

All the Llama 3 outputs for each of the 3 temperatures matched the required output (rating separate from an explanation) for each of the 5 prompts for all 180 questions and were included in the analysis.

### Concordance

**Fig. 3:**
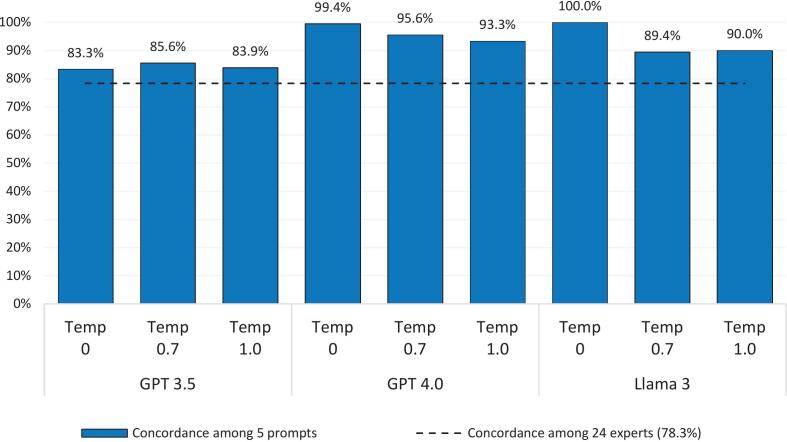
Concordance by LLM and temperature.

In the original Delphi study, the 24 human experts reached concordance on 141 of the 180 questions (78.3%). Overall concordance for each LLM and temperature are shown in Fig. 3 along with the expert human concordance.

Each LLM and temperature combination had higher concordance than the human experts.

Table 3 shows the concordance of the five prompts per LLM with the expert human concordance (Temp = temperature setting).

**Table 3:** Concordance of LLM and human experts.

Concordance (IQR ≤1)			Experts				Total	% of total (*n* = 180)
			Yes	% of total (*n* = 180)	No	% of total (*n* = 180)		
GPT 3.5	Temp 0	Yes	115	63.9%	35	19.4%	150	83.3%
		No	0	0.0%	0	0.0%	0	0.0%
		Unknown^a^	26	14.4%	4	2.2%	30	16.7%
	Temp 0.7	Yes	120	66.7%	34	18.9%	154	85.6%
		No	5	2.8%	2	1.1%	7	3.9%
		Unknown^a^	16	8.9%	3	1.7%	19	10.6%
	Temp 1.0	Yes	118	65.6%	33	18.3%	151	83.9%
		No	11	6.1%	2	1.1%	13	7.2%
		Unknown^a^	12	6.7%	4	2.2%	16	8.9%
GPT 4.0	Temp 0	Yes	140	77.8%	39	21.7%	179	99.4%
		No	0	0.0%	0	0.0%	0	0.0%
		Unknown^a^	1	0.6%	0	0.0%	1	0.6%
	Temp 0.7	Yes	134	74.4%	38	21.1%	172	95.6%
		No	7	3.9%	1	0.6%	8	4.4%
	Temp 1.0	Yes	131	72.8%	37	20.6%	168	93.3%
		No	8	4.4%	2	1.1%	10	5.6%
		Unknown^a^	2	1.1%	0	0.0%	2	1.1%
Llama 3	Temp 0	Yes	141	78.3%	39	21.7%	180	100.0%
		No	0	0.0%	0	0.0%	0	0.0%
	Temp 0.7	Yes	124	68.9%	37	20.6%	161	89.4%
		No	17	9.4%	2	1.1%	19	10.6%
	Temp 1.0	Yes	126	70.0%	36	20.0%	162	90.0%
		No	15	8.3%	3	1.7%	18	10.0%
Total			141	78.3%	39	21.7%	180	100.0%

a Unknown due to IQR being unable to be calculated because of missing rating or only single prompt provided rating.

Overall, LLM concordance in cases where human experts were not concordant ranged from *n* = 33 (18.3%, GPT 3.5 Temp 1.0) to *n* = 39 (21.7%, GPT 4.0 Temp 0, and Llama 3 Temp 0). Overall, LLM discordance in cases where human experts were concordant ranged from *n* = 0 (0%, GPT 3.5 Temp 0, GPT 4.0 Temp 0, and Llama 3 Temp 0) to *n* = 17 (9.4%, Llama 3 Temp 0.7).

**Fig. 4:**
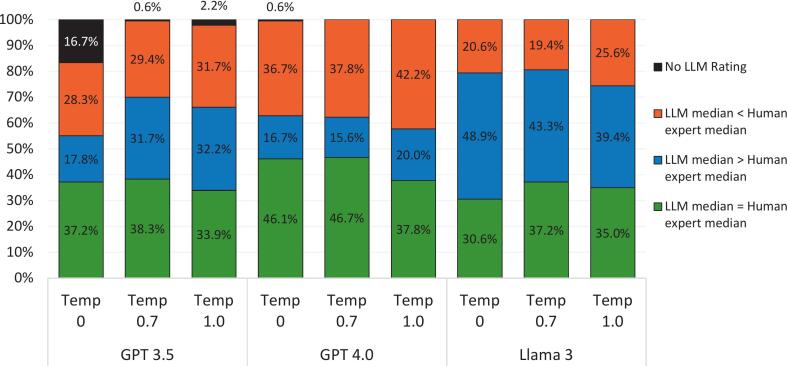
Proportion of medians higher, lower, equal between LLM and human experts.

Fig. 4 shows the proportion of cases where the median was higher, lower, or equal for LLM versus human experts.

The LLMs with the highest volume of equivalent median ratings to humans were GPT 4.0 Temp 0.7 (46.7%) and GPT 4.0 Temp 0 (46.1%) assessments. Llama 3 had the highest proportion of ratings with higher median ratings for LLM than humans (48.9% Llama 3 Temp 0, 43.3% Llama 3 Temp 0.7). GPT 4.0 Temp 1.0 had the highest proportion of cases where humans provided higher median ratings than LLM (42.2%).

**Fig. 5:**
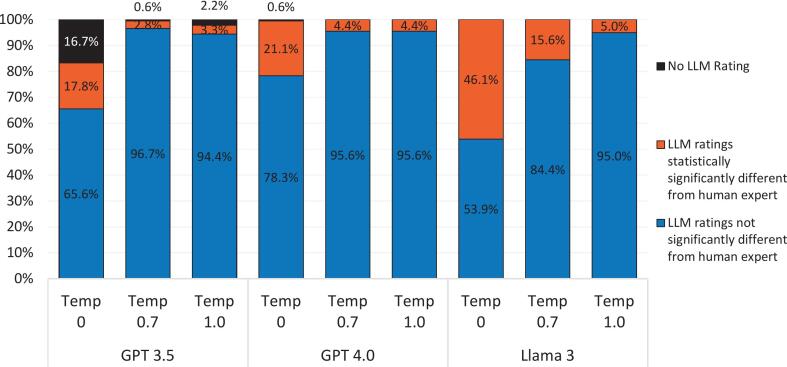
Proportion of cases with statistically significantly different or equivalent medians.

Fig. 5 shows the proportion of cases where the median was statistically significantly different between LLM and expert humans.

Llama 3 Temp 0 ratings were significantly different from humans for 46.1% of the questions asked. Fewer cases were significantly different between LLM and humans for GPT 3.5 Temp 0.7 (2.8%) and GPT 3.5 Temp 1.0 (3.3%).

## Discussion

This study represents, to the authors' knowledge, the first instance of a Delphi study in medicine being conducted solely using AI to perform the job normally allocated to a human expert panel. In this study, the three LLM models ChatGPT 3.5, ChatGPT 4.0, and Llama 3, were put to the test to see how well they performed at this task relative to the results already previously collected from a human expert panel regarding the future of Computational Pathology and AI for a 2023 Delphi study published in *eBioMedicine*.^1^ The results demonstrate several key takeaways. Firstly, in terms of the fidelity of matching the required formatting and outputting a numerical Likert rating separate from the textual explanation, Llama 3 performed the best, matching the correct format in every instance without any manual recoding efforts required. Meanwhile, ChatGPT 4.0 failed to automatically output the desired formatting and separate numerical Likert rating in some instances. However, with manual recoding efforts, ChatGPT 4.0 only had one instance at temperature 0, where it provided no numerical Likert rating at all, even within the textual explanation, making it impossible to recode. All other temperature/question combinations for ChatGPT 4.0 were rectifiable to the correct output format via manual recoding. Furthermore, for ChatGPT 4.0, at temperature 0 specifically, only 1.7% of cases (3 out of 180) required any manual recoding at all, and of those, 66.7% (2 out of 3) were successfully recoded from the initial AI formatting missing a numerical rating. At higher temperatures for ChatGPT 4.0 (0.7 and 1.0), although a greater number of questions required manual recoding (67 items for temperature 0.7 and 98 items for temperature 1.0), there were also no recoding issues; meaning, both temperatures 0.7 and 1.0 for ChatGPT 4.0 had a successful recoding rate of 100%. In contrast, ChatGPT 3.5 required the most manual recoding across all temperature settings, with 56.1% of questions (101 items) needing recoding at temperature 0, 71.1% of questions (128 items) at temperature 0.7, and 76.7% of questions (138 items) at temperature 1.0. Whereas ratings from ChatGPT-3.5 were generally provided in the rationale in most cases, 30 of the 101 issues at temperature 0 (29.7%), 1 of the 128 issues at temperature 0.7 (0.8%), and 4 of the 138 issues at temperature 1.0 (2.9%), were not able to be recoded due to the complete lack of an extractable numerical Likert rating.

Clearly, the burden of recoding work required of the human researchers increased in the order of Llama 3 being the least with no manual recoding required, followed by ChatGPT 4.0, and then ChatGPT 3.5, which necessitated the greatest manual recoding effort. The impact of temperature on recoding frequency is also notable, as higher temperatures (such as 0.7 and 1.0) led to an increase in the number of cases requiring recoding for both ChatGPT 4.0 and ChatGPT 3.5. Whereas for ChatGPT 4.0, we successfully recoded 100% of cases at both higher temperatures, the overall burden of recoding still increased due to the larger number of cases that required manual intervention at these temperatures. This suggests that lower temperature settings may lead to more precisely formatted outputs per researcher specifications, whereas higher temperatures introduce more variability and a greater likelihood of requiring manual intervention, albeit potentially leading to a full recoding success rate.

It is imperative, however, to balance minimization of the manual recoding burden faced by the human researchers with the output quality provided by each model, in terms of both concordance reached among AI trials for each question item, and its agreement with the human expert control set, as the accuracy, conclusiveness, and relevance of the ratings provided by the model play the most crucial role in the utility of the data and findings of a Delphi study. In this domain, the clear takeaway is that the specific LLM, as well as the temperature setting, that one chooses really does fundamentally impact the AI inter-trial concordance, as well as the level of agreement of AI's results with those of a human expert panel validation control set. Out of the 180 questions, ChatGPT-3.5 across the 3 temperature settings reached consensus for only between 83.3 and 85.6% of the 180 items. Meanwhile, Llama 3 had 100% concordance (for all 180 question items) at temperature 0, meaning it reached a consensus among the 5 trials for each of the 180 questions; however, it dropped to 89.4% of questions reaching consensus at temperature 0.7 and 90.0% at temperature 1.0. As for ChatGPT-4.0, at temperature 0, it was able to come to a consensus answer for 99.4% of the 180 questions, and this stayed steadier as temperature increased for ChatGPT-4.0, finding concordance for 95.6% of questions at temp 0.7 and 93.3% at temp 1.0. This makes sense and shows us that the model we pick greatly matters, as ChatGPT-4.0 is a key industry leader, whereas Llama 3 is a high-quality model but nevertheless less capable open source alternative, and ChatGPT-3.5 is simply an older model included in the study for illustrating this exact point, and the level of concordance directly correlates to the generally perceived “quality” of each model. Importantly, higher concordance among model-generated responses should not be interpreted as evidence of greater epistemic validity, but rather as a reflection of the internal consistency of the model's generative process compared with the heterogeneity inherent in human expert panels.

Whereas variability existed across all temperature/model combinations, a general trend emerged: at higher temperature settings, LLMs appeared to make more predictions that were statistically closer to human expert opinions (based on comparisons of median ratings as seen in Fig. 5). This could be due to increased creativity allowing the LLMs to better approximate the nuance and implicit judgment that human experts bring to ambiguous or predictive questions. Interestingly, in some instances, the LLMs were more optimistic about AI adoption timelines than the human panel, likely reflecting AI's reliance on aspirational themes prevalent in published literature rather than practical limitations known to domain experts. Furthermore, we clearly see here that we are more likely to get concordant results for more of the questions items when we lower the temperature setting on the LLM. As demonstrated, temperature controls randomness in the responses of a LLM. A lower temperature (closer to 0) makes the model more deterministic, meaning it sticks to the most likely answer and avoids unexpected variations. This is great when precision matters, like when answering factual questions. A higher temperature (closer to 1) increases randomness, encouraging the model to take risks and generate more creative or diverse responses. This is helpful for brainstorming, storytelling, or open-ended conversations where originality is key.

However, thinking about the agreement of the AI findings with those of the human expert panel which was treated as a control for validation here, whereas Fig. 3 demonstrates variability across the board in terms of which question items were given empirically higher, lower, or equivalent median Likert rankings to those generated by the expert humans, in Fig. 4 we see that across all LLM models, increasing temperature (which allows for greater creativity) dramatically improves agreement level between AI's predictions and those of the human expert panel control (when comparing the difference between them on the basis of statistical significance). This demonstrates that allowing AI a greater amount of freedom and creativity in its response generation process led to more realistic AI outputs than (or statistically equivalent to) those which would be outputted by human experts. Thus, there is a clear trade-off between concordance (i.e., consensus among answers to the same question across the trials performed for that question), which is of great importance in a Delphi study because concordance must be reached among the responses to any given item in order to be able to conclude anything about that item, and the quality of the output itself, for we cannot simply prioritize only concordance at the complete expense of having a realistic and meaningful nature of those outputs. Especially since, after all, the goal is to produce outputs that accurately emulate those which might be produced by a hypothetical human expert panel. Today, there are several LLMs. However, for the purpose of this study, only three were selected. One caveat is that the human Delphi study was undertaken several years ago. The accumulation of information since then has been remarkable and could thus contribute to the reduced consensus. Further, it is plausible that the LLMs had access to the original open access Delphi study, and as such this data leakage may have introduced bias.

From this, we clearly see that there is a multi-faceted balance that must be struck between concordance, answer quality and realism, and manual recoding burden placed upon the researchers, a balance which is determined by the combination of LLM and temperature setting chosen. In turn, there is no clear cut “best combination” of model and temperature setting across every Delphi study. For example, if the goal of a particular Delphi project is simply to generate a consensus of what current evidence states about a specified topic because such data already exist but simply has never been aggregated into an overarching idea of what experts on the whole think before, then a lower temperature setting may be best to ensure that all lines of current evidence are synthesized as accurately as possible into a consensus finding. Whereas if the goal of another Delphi study is to try to generate novel ideas to give researchers in a field food-for-thought regarding creative new research directions that may have never been considered before even by the experts (i.e., a more exploratory rather than summatory aim), then a higher temperature to allow the LLM more creativity and novel experimentation towards producing its outputs may be worth the sacrifice of a small decrease in the amount of question outputs which would putatively agree with an equivalent real human expert panel's consensus findings, should such a comparison be hypothetically undertaken.

Thus, in reality, whereas AI can help dramatically expedite the number of Delphi studies that can be conducted by the medical community and the rate at which they can be performed and disseminated, still, there will at least for the foreseeable future continue to be a necessary degree of human oversight for these studies in regards to applying intuition in choosing the most appropriate model and temperature combination for a given Delphi study and its unique aims, deciding what balance to strike in terms of concordance, manual recoding burden, and the likely realism of the results if they were compared to a control expert panel (hypothetically speaking, as by design, future AI-run Delphi studies will not necessarily have a parallel human-conducted study to compare to anymore).

Because the original Delphi questions were reformulated for compatibility with the Delphi-GPT framework, semantic fidelity between the original and reformulated questions was assessed manually by the authors using native English-language proficiency. This process may introduce a small degree of subjective judgment and highlights the need for future work exploring more standardized or automated approaches to validating semantic equivalence.

Further, as a pilot proof-of-concept study, these findings primarily demonstrate the general feasibility of using a structured prompting framework to simulate Delphi-style consensus generation with a LLM. Whereas the observed relationships between temperature, response variability, and consensus provide useful initial insights, any recommendations regarding specific model or temperature configurations should be interpreted as preliminary. Further validation across additional medical domains, question types, and expert consensus tasks will be necessary before such configurations could be considered broadly generalizable.

It is essential to underscore that whereas LLMs can mimic aspects of human consensus, they do not entirely replace the process of novel human dialog, deliberation, and experiential judgment in situations where these aspects of the study are invaluable. Whereas running the Delphi method with an AI panel may be far more empirically evidence-based given its access to a vast ocean of training data, a simulated agreement derived from LLMs will likely always lack the iterative social learning and interpretive depth that can come from real-time expert discussion. This limitation should be kept in mind when interpreting any AI-generated Delphi findings and when determining whether a human panel is irreplaceable for any given Delphi study based on its specific goals. Thus, this study does not advocate for replacing human experts with autonomous LLMs altogether. Instead, it positions LLM Delphi panels as powerful but complementary tools, which do not replace the niche of the human Delphi panel, but which may be the more appropriate tool to apply to a given study based on its particular goals. Thus, it is unlikely that an AI-run Delphi will fully supplant the contextual wisdom, adaptability, and accountability that human panels provide; rather, these may come to be two equally invaluable separate assets to the research community, fulfilling two different niches within the overall realm of the Delphi method, which in turn will hopefully allow for a greater diversity of more robust studies to be performed, leading us to new medical insights faster. Additionally, because of this study, there now exists an easy implementation solution for conducting further AI-run Delphi studies in medical research, the “Delphi-GPT” framework as developed here. To facilitate the ability of future research teams to complete AI-based Delphi studies, whether total or hybrid, we have made freely available, in the supplementary materials accompanying this article on the journal website, the code for the Delphi-GPT program, and permission to modify the code to work with additional LLMs or features of one's own choosing, as long as proper credit is given to this original study.

One important limitation of AI-run Delphi studies to note is that all the outputs of an LLM are based upon pre-existing training data, or in other words, it cannot produce any purely novel ideas that are completely independent of any previously existing information. That is to say, it lacks true innate human intuition. Still, considering that much of these training data would come from sources written by the same experts who may be consulted on Delphi study panels, and the fact that the LLMs are able to pull upon various lines of previous information to synthesize ideas, which are themselves a novel conglomeration despite being derived wholly from previously published lines of evidence, a lack of “pure” originality from an incapacity for intuitive, rather than evidence-based, thought does not necessarily preclude AI from being able to produce meaningful predictions and insights for Delphi studies. In fact, AI may be better suited for running Delphi studies in this regard as compared to human experts, who bring bias and subjectivity into the equation, whereas LLMs can simply evaluate all previously existing lines of relevant evidence, and synthesize them objectively into the most statistically likely prediction or insight, based on what is known and nothing more, outputs which can get progressively more creative and novel the higher the temperature setting is set. Still, it should be re-emphasized that LLMs are dependent on historical context, whereas human experts are able to apply intuition, judgment, and lived clinical experience in real time. These human capabilities remain an indispensable tool, especially in high-stakes settings, or when tackling emerging under-documented challenges for which an autonomous AI Delphi panel would surely not yield meaningful results. Furthermore, addressing potential biases in AI outputs due to reliance on pre-existing training data within the LLM itself remains a limitation at present which needs to be addressed moving forward.

Another limitation of the present study is that no retrieval-augmented generation (RAG) framework was employed, nor was the retrieval layer filtered to restrict access to information that existed only before the date on which the original human Delphi study was conducted. Similarly, no prompt-level filtering was implemented to prevent the LLMs from referencing material that post-dated the target study period. As such, it is possible that some of the outputs generated by the LLMs may have been informed by developments, publications, or discourse that occurred after the original expert panel concluded its work. This temporal mismatch may introduce bias favoring the LLMs by allowing them to benefit from hindsight or later consensus that would not have been available to human experts at the time. LLMs, in general, may have access to literature published after an original Delphi study used as a human comparison benchmark, which could introduce a potential informational asymmetry when comparing AI-generated responses with historical human consensus in future proof-of-concept studies building upon the present work. However, in prospective applications of a framework such as applying Delphi-GPT to newly conducted Delphi studies post-validation, both human experts and AI systems would draw from the same existing body of prior literature available at the time of the study. In this context, the primary difference would likely lie in the scale and speed with which AI systems can process available information rather than access to fundamentally different sources of knowledge, which may represent an efficiency advantage of AI over human experts when applying frameworks such as Delphi-GPT to de novo Delphi studies in medicine. Future studies aiming to benchmark AI performance against historical human panels should consider restricting the information access window accordingly to ensure a fairer comparison.

Additionally, the definition of consensus used in this study (IQR ≤ 1) was selected to mirror the methodology of the original Delphi study used as the comparison benchmark; however, it represents one of several possible approaches to defining agreement in Delphi methodology and may not fully capture all dimensions of consensus. Furthermore, only a single historical Delphi study was used as the reference for comparison, which may limit generalizability and result in a relatively small effective sample size. As such, findings should be interpreted within the context of a pilot proof-of-concept study and require validation across additional datasets and clinical domains.

We wish to briefly take the opportunity here to additionally provide the thoughts of AI itself regarding the key differences between traditional Delphi studies and Delphi studies run by LLMs, and when one might consider using one or the other. This can be found in Table 4 below.

**Table 4:** The thoughts of AI itself on differences between and use cases for traditional Delphi studies versus LLM-based Delphi studies.

Reaching consensus: Traditional Delphi vs LLM		
Aspect	Traditional Delphi Study	LLM (e.g., GPT-4)
Participants	Real human experts	Trained on expert-written text
Process	Iterative, anonymized rounds	Single or multi-turn interaction
Consensus	Emerges through real expert alignment	Simulated via pattern recognition and synthesis
Bias Control	Designed to reduce groupthink	Subject to training data biases
Transparency	High (you can track responses and revisions)	Lower (black-box reasoning, not always explainable)
Speed & Scalability	Slow, small sample	Fast, highly scalable
Contextual Expertise	Deep domain knowledge and justification	Generalist, sometimes shallow without fine-tuning
Validation	Peer-reviewed or referenced	Requires human validation

When LLMs might be “better” Speed and cost-efficiency: LLMs can rapidly generate or synthesize consensus-like answers. Early-stage idea generation: They can help formulate initial frameworks or identify areas of agreement/disagreement. Simulating multiple viewpoints: LLMs can be prompted to imitate various expert stances.		

When traditional Delphi is preferable High-stakes decisions: When consensus must be validated by real experts. Novel topics: Where LLMs may lack updated or nuanced domain-specific knowledge. Accountability and transparency: Delphi provides traceable expert input.		

* Table 4 modified from direct output of ChatGPT-4.0.

Looking towards the future, as progress in the field of AI continues to catapult forward with rapid speed, surely new LLMs and/or newer iterations of the LLMs used in this study will supplant the ones used here. However, more important than any of the specifics of the models used here, is the proof-of-concept. All the models and temperature settings used here, even the worst performing ones, found greater concordance in their predictions than a real panel of human experts could. Thus, regardless of the models that will be used in a year from now, 5 years from now, or even a decade from now, the results here broadly demonstrate that running a medical Delphi study with LLMs taking on the role of the expert panel is already feasible, given some human oversight such as the need for manually recoding incorrectly formatted initial outputs and selecting the most appropriate model/temperature combination for the aims of the specific Delphi study at hand. As newer, more capable LLMs continue to reach the market, their ability to conduct high-quality medical Delphi studies will only continue to improve and be validated by further such Delphi studies, and the “Delphi-GPT” program as provided here creates a flexible baseline framework upon which to continue to streamline the conducting of medical Delphi studies as we move forward, as it can continue to be modified for usage with better LLMs of the future.

## Declaration of competing interest

The authors declare the following financial interests/personal relationships which may be considered as potential competing interests:

Christopher Daniel Manko reports a relationship with DataAnnotation Tech that includes: employment. Colleen Vrbin reports a relationship with Analytical Insights, LLC that includes: board membership, consulting or advisory, employment, and equity or stocks. Jochen K. Lennerz reports a relationship with BostonGene Corporation that includes: employment. Liron Pantanowitz reports a relationship with AIxMed Inc. that includes: consulting or advisory. Liron Pantanowitz reports a relationship with Hamamatsu Photonics KK that includes: consulting or advisory. Liron Pantanowitz reports a relationship with NTP Nano Tech Projects S.r.l. that includes: consulting or advisory and equity or stocks. Liron Pantanowitz reports a relationship with Ibex Medical Analytics Ltd. that includes: board membership and equity or stocks. Liron Pantanowitz reports a relationship with Placenta AI that includes: equity or stocks. Liron Pantanowitz reports a relationship with LeanAP that includes: equity or stocks. Jeanne Shen reports a relationship with Yari Care, Inc. that includes: consulting or advisory. Jeanne Shen reports a relationship with National Institutes of Health that includes: funding grants. Jeanne Shen reports a relationship with Lunit Inc. that includes: funding grants. Jeanne Shen reports a relationship with Roche Diagnostics Corporation that includes: speaking and lecture fees. Hooman Rashidi reports a relationship with MILO-ML LLC that includes: board membership and equity or stocks. Hooman Rashidi reports a relationship with STNG that includes: equity or stocks.

Liron Pantanowitz - Editor in Chief of JPI. Hooman Rashidi - Associate Editor of JPI. David McClintock - Editorial Board Member of JPI. Jeroen Van der Laak - Editorial Board Member of JPI. J. Mark Tuthill - Editorial Board Member of JPI. Jerome Y. Cheng - Editorial Board Member of JPI. Jochen K. Lennerz - Editorial Board Member of JPI. John H. Sinard - Editorial Board Member of JPI. Mohamed E. Salama - Editorial Board Member of JPI.

Given their role as editors of JPI, all of the above authors had no involvement in the peer review of this article and had no access to information regarding its peer review. Full responsibility for the editorial process for this article was delegated to another journal editor. If there are other authors, they declare that they have no known competing financial interests or personal relationships that could have appeared to influence the work reported in this article.

## Data Availability

In addition to the data and statistical figures as presented within the body of the article, the raw data and statistical analyses outputted by the LLM models are included in the Appendix and supplementary materials accompanying this manuscript on the journal website. The code for the Delphi-GPT model created and used for the study is also shared in the supplementary material. Furthermore, any data used from the original 2023 article in the journal *eBioMedicine* upon which the present article is based are accessible in the body of that original article and/or its Appendix and supplementary materials published as Open Source, found online at the following URL: https://doi.org/10.1016/j.ebiom.2022.104427 (and that article is included among the references for the present article). Permission is given for further usage and modification of the Delphi-GPT code so long as proper credit is given to the original article for providing the source code and any published research works using it appropriately cite this article. Please note that neither the authors, nor any academic journals, publishers of journals, or any other possibly related entities or subsidiaries, are responsible for any fees or other consequences incurred as a result of further usage or application of the findings/data/code/other materials, as shared here, towards any further research or other endeavors.

## Appendix A Supplementary data

Supplementary material 1 Original data used in the study and code for the Delphi-GPT application.
