# Supplementary material for: How does AI perform compared to human expert panels in medical Delphi studies? A pilot study through the lens of pathology
Source: J Pathol Inform. 2026 Apr 15;21:100661. doi: 10.1016/j.jpi.2026.100661 (PMC13185839; doi:10.1016/j.jpi.2026.100661)
Supplement: Supplementary material 1 — Original data used in the study and code for the Delphi-GPT application. [file mmc1.zip › Delphi-Results-&-Code-Sent-to-copy/Documenation & Codes-for-each-of-the-Category-runs/Delphi-App-documentation.docx]

Delphi Code Explanation:

# Delphi-App-Script by Hooman H. Rashidi

**Note: Hope you find it helpful but no support or guarantee provided on the use of this code**

## Introduction

This script is designed to automate the process of collecting expert ratings on specific statements regarding the future of AI in pathology by 2030. It uses OpenAI's API to generate ratings and explanations for each statement in a CSV file.

## Setup

### Dependencies

- openai

- pandas

- csv

You can install the necessary Python packages using pip:

```bash

pip install openai pandas

**----------------------------------------------------------------------------------**

**OpenAI API Key**

You need to have an OpenAI API key to use the script. Replace 'your openai api key goes here' with your actual API key. Make sure to keep your API key private.

**Script Explanation**

**Importing Libraries**

The script starts by importing the required libraries: openai for accessing the OpenAI API, pandas for handling data in DataFrames, and csv for CSV file operations.

python

Copy code

from openai import OpenAI

import pandas as pd

import csv

**Setting Up OpenAI API Client**

The script initializes the OpenAI API client with the provided API key.

python

Copy code

client = OpenAI(api_key='your openai api key goes here') # Replace with your actual API key (and make sure you don't share it so that it stays private)

**Loading the CSV File**

The input and output file paths are defined. The input file is loaded into a pandas DataFrame.

python

Copy code

input_file_path = './Category-7.csv'

output_file_path = './completed-ai-survey7-GPT4o-temp00-w-avg.csv'

df = pd.read_csv(input_file_path, encoding='latin1')

**Defining the Prompt Template (as shown in the example below)**

A prompt template is defined to instruct the AI model on how to rate each statement and provide explanations.

python

Copy code

prompt_template = """

In this round, you will be asked to rate each of the specified statements provided to you from the CSV file according to the Likert scale below. You will rate each specified statement one trial at a time, one specified statement per trial (i.e. one trial at a time, with each trial regarding only one specified statement).

Please answer considering only AI input, not digital pathology in a broad sense. Also, please answer according to what you believe will happen by 2030, instead of what you would like to happen.

All of your responses will remain anonymous to the rest of the panel experts.

Please estimate the PROBABILITY that this task will become FULLY DELEGATED to AI and thus done in a FULLY AUTOMATED WAY in pathology labs by 2030, by selecting one of the following Likert scale responses with which to complete the specified statement: Note that these are in 10% increments meaning that 0.1 is 10%, 0.2 is 20%, all the way to 1 which is 100%.

1 Impossible (0)

2 Very unlikely (0 - 0.2)

3 Unlikely (0.2 - 0.4)

4 Even chance/neutral (0.4 - 0.6)

5 Likely (0.6 - 0.8)

6 Very likely (0.8 - 1)

7 Certain (1)

You may only rank based on these discrete categories (1,2,3,4,5,6, or 7), no in-betweens are acceptable. Further, giving two or more Likert rankings to avoid definitively deciding is not allowed; when in doubt, you must provide your best single Likert ranking, and instead you may acknowledge any difficulties you had in coming to a final single Likert ranking within your follow-up rationale/explanation, but still you must provide a single discrete Likert ranking as your final answer, as this data will ultimately be entered categorically into an Excel file.

Please additionally provide a rationale/explanation behind why you decided to give the ranking you did.

Specified statement: "{statement}"

"""

**Function to Get Ratings and Explanations**

The function below is defined to get five ratings and explanations from the different Open AI GPT models for each statement as shown in the example below for GPT-4omni (GPT-4o)

python

Copy code

def get_gpt4_ratings(statement):

prompt = prompt_template.format(statement=statement)

response = client.chat.completions.create(

model="gpt-4o",

messages=[

{"role": "system", "content": "You are a helpful assistant."},

{"role": "user", "content": prompt}

],

max_tokens=200,

n=5, # Request 5 completions

temperature=0

)

ratings = []

explanations = []

for choice in response.choices:

response_text = choice.message.content.strip()

lines = response_text.split('\n')

rating = lines[0].strip()

rating_value = ''.join(filter(str.isdigit, rating))

# Validate rating and ensure it is within the range 1-7

if rating_value.isdigit() and 1 <= int(rating_value) <= 7:

ratings.append(int(rating_value))

else:

ratings.append(None) # Mark invalid ratings as None

explanation = '\n'.join(lines[1:]).strip()

explanations.append(explanation if explanation else "No explanation provided")

return ratings, explanations

**Creating Lists to Store Results**

Lists are created to store the ratings and explanations for each statement.

python

Copy code

ratings_list = [[] for _ in range(5)]

explanations_list = [[] for _ in range(5)]

**Iterating Through Each Statement**

The script iterates through each statement in the DataFrame, gets the ratings and explanations, and stores them in the respective lists.

python

Copy code

for statement in df['Specified Statement']:

ratings, explanations = get_gpt4_ratings(statement)

# Ensure all ratings are valid and not None

ratings = [r if r is not None else "Invalid" for r in ratings]

for i in range(5):

ratings_list[i].append(ratings[i])

explanations_list[i].append(explanations[i])

**Adding Ratings and Explanations to DataFrame**

The ratings and explanations are added to the DataFrame as new columns.

python

Copy code

for i in range(5):

df[f'AI Rating {i+1}'] = ratings_list[i]

df[f'AI Explanation {i+1}'] = explanations_list[i]

**Calculating Average Ratings**

The average rating for each row is calculated, ignoring invalid ratings.

python

Copy code

df['Average AI Ratings'] = df[[f'AI Rating {i+1}' for i in range(5)]].replace("Invalid", None).astype(float).mean(axis=1)

**Saving the Completed DataFrame**

The completed DataFrame is saved to a new CSV file.

python

Copy code

df.to_csv(output_file_path, index=False, quoting=csv.QUOTE_NONNUMERIC)

print(f"Completed survey saved to {output_file_path}")

**Conclusion**

This script provides a method to automate the collection of expert ratings on specific statements regarding the future of AI in pathology. By using OpenAI's API, it generates multiple ratings and explanations, calculates the average rating, and saves the results in a new CSV file.
