# Supplementary material for: How does AI perform compared to human expert panels in medical Delphi studies? A pilot study through the lens of pathology
Source: J Pathol Inform. 2026 Apr 15;21:100661. doi: 10.1016/j.jpi.2026.100661 (PMC13185839; doi:10.1016/j.jpi.2026.100661)
Supplement: Supplementary material 1 — Original data used in the study and code for the Delphi-GPT application. [file mmc1.zip › Delphi-Results-&-Code-Sent-to-copy/original-excel-files 1-7 & word file 1-7 prompt/General Prompts Doc.docx]

***Category 1 Prompt:***

This is part of a study about what the future of AI in pathology will look like by 2030. The expert answers to Round 1 have been transformed into a series of statements, which will now be assessed by 1) humans and 2) AI models like yourself. In this round, you will be asked to rate each of the specified statements provided to you from the CSV file according to the Likert scale below. You will rate each specified statement one trial at a time, one specified statement per trial (i.e. one trial at a time, with each trial regarding only one specified statement).

Please answer considering only AI input, not digital pathology in a broad sense. Also, please

answer according to what you believe will happen by 2030, instead of what you would like to

happen.

All of your responses will remain anonymous to the rest of the panel experts.

Please rate your AGREEMENT with the specified statement according to this Likert scale:

1 Very strongly disagree

2 Strongly disagree

3 Disagree

4 Neither agree nor disagree

5 Agree

6 Strongly agree

7 Very strongly agree

You may only rank based on these discrete categories (1,2,3,4,5,6, or 7), no in-betweens are acceptable. Further, giving two or more Likert rankings to avoid definitively deciding is not allowed; when in doubt, you must provide your best single Likert ranking, and instead you may acknowledge any difficulties you had in coming to a final single Likert ranking within your follow-up rationale/explanation, but still you must provide a single discrete Likert ranking as your final answer, as this data will ultimately be entered categorically into an Excel file.

Please additionally provide a rationale/explanation behind why you decided to give the ranking you did.

Here is the specified statement you are ranking according to the Likert scale for this trial: 

***“Insert Statement Here (via Coded Script)”***

***Category 2 Prompt:***

This is part of a study about what the future of AI in pathology will look like by 2030. The expert answers to Round 1 have been transformed into a series of statements, which will now be assessed by 1) humans and 2) AI models like yourself. In this round, you will be asked to rate each of the specified statements provided to you from the CSV file according to the Likert scale below. You will rate each specified statement one trial at a time, one specified statement per trial (i.e. one trial at a time, with each trial regarding only one specified statement).

Please answer considering only AI input, not digital pathology in a broad sense. Also, please

answer according to what you believe will happen by 2030, instead of what you would like to

happen.

All of your responses will remain anonymous to the rest of the panel experts.

Please estimate how the integration of AI in the Pathology setting will impact the workforce, by selecting one of the following Likert scale responses with which to complete the specified statement:

1 Dramatically decrease (from -50% to -100%)

2 Greatly decrease (from -20% to -50%)

3 Somewhat decrease (from -5% to -20%)

4 Remain the same (from -5% to +5%)

5 Somewhat increase (from +5% to +20%)

6 Greatly increase (from +20% to +50%)

7 Dramatically increase (from +50% to +100%)

You may only rank based on these discrete categories (1,2,3,4,5,6, or 7), no in-betweens are acceptable. Further, giving two or more Likert rankings to avoid definitively deciding is not allowed; when in doubt, you must provide your best single Likert ranking, and instead you may acknowledge any difficulties you had in coming to a final single Likert ranking within your follow-up rationale/explanation, but still you must provide a single discrete Likert ranking as your final answer, as this data will ultimately be entered categorically into an Excel file.

Please additionally provide a rationale/explanation behind why you decided to give the ranking you did.

Here is the specified statement which you are completing by filling in the end with your chosen Likert-ranked option for this trial: 

***“Insert Statement Here (via Coded Script)”***

***Category 3 Prompt:***

This is part of a study about what the future of AI in pathology will look like by 2030. The expert answers to Round 1 have been transformed into a series of statements, which will now be assessed by 1) humans and 2) AI models like yourself. In this round, you will be asked to rate each of the specified statements provided to you from the CSV file according to the Likert scale below. You will rate each specified statement one trial at a time, one specified statement per trial (i.e. one trial at a time, with each trial regarding only one specified statement).

Please answer considering only AI input, not digital pathology in a broad sense. Also, please

answer according to what you believe will happen by 2030, instead of what you would like to

happen.

All of your responses will remain anonymous to the rest of the panel experts.

Please estimate the degree of involvement of pathologists in the task by 2030, by selecting one of the following Likert scale responses with which to complete the specified statement:

1 Not involved at all

2 Rarely involved

3 Somewhat involved

4 Sometimes involved

5 Often involved

6 Routinely involved

7 Involved daily

You may only rank based on these discrete categories (1,2,3,4,5,6, or 7), no in-betweens are acceptable. Further, giving two or more Likert rankings to avoid definitively deciding is not allowed; when in doubt, you must provide your best single Likert ranking, and instead you may acknowledge any difficulties you had in coming to a final single Likert ranking within your follow-up rationale/explanation, but still you must provide a single discrete Likert ranking as your final answer, as this data will ultimately be entered categorically into an Excel file.

Please additionally provide a rationale/explanation behind why you decided to give the ranking you did.

Here is the specified statement which you are completing by filling in the end with your chosen Likert-ranked option for this trial: 

***“Insert Statement Here (via Coded Script)”***

***Category 4 Prompt:***

This is part of a study about what the future of AI in pathology will look like by 2030. The expert answers to Round 1 have been transformed into a series of statements, which will now be assessed by 1) humans and 2) AI models like yourself. In this round, you will be asked to rate each of the specified statements provided to you from the CSV file according to the Likert scale below. You will rate each specified statement one trial at a time, one specified statement per trial (i.e. one trial at a time, with each trial regarding only one specified statement).

Please answer considering only AI input, not digital pathology in a broad sense. Also, please

answer according to what you believe will happen by 2030, instead of what you would like to

happen.

All of your responses will remain anonymous to the rest of the panel experts.

Please estimate the degree of involvement of pathology technicians in the task by 2030, by selecting one of the following Likert scale responses with which to complete the specified statement:

1 Not involved at all

2 Rarely involved

3 Somewhat involved

4 Sometimes involved

5 Often involved

6 Routinely involved

7 Involved daily

You may only rank based on these discrete categories (1,2,3,4,5,6, or 7), no in-betweens are acceptable. Further, giving two or more Likert rankings to avoid definitively deciding is not allowed; when in doubt, you must provide your best single Likert ranking, and instead you may acknowledge any difficulties you had in coming to a final single Likert ranking within your follow-up rationale/explanation, but still you must provide a single discrete Likert ranking as your final answer, as this data will ultimately be entered categorically into an Excel file.

Please additionally provide a rationale/explanation behind why you decided to give the ranking you did.

Here is the specified statement which you are completing by filling in the end with your chosen Likert-ranked option for this trial:

***“Insert Statement Here (via Coded Script)”***

***Category 5 Prompt:***

This is part of a study about what the future of AI in pathology will look like by 2030. The expert answers to Round 1 have been transformed into a series of statements, which will now be assessed by 1) humans and 2) AI models like yourself. In this round, you will be asked to rate each of the specified statements provided to you from the CSV file according to the Likert scale below. You will rate each specified statement one trial at a time, one specified statement per trial (i.e. one trial at a time, with each trial regarding only one specified statement).

Please answer considering only AI input, not digital pathology in a broad sense. Also, please

answer according to what you believe will happen by 2030, instead of what you would like to

happen.

All of your responses will remain anonymous to the rest of the panel experts.

Please estimate the PROBABILITY of this application of AI being used routinely in pathology labs by 2030, by selecting one of the following Likert scale responses below with which to complete the specified statement. Remember, you are ranking the probability of the task in the specified statement being specifically performed with/by application of AI, not the probability of whether the task will be performed in general.

1 Impossible (0)

2 Very unlikely (0 - 0.2)

3 Unlikely (0.2 - 0.4)

4 Even chance/neutral (0.4 - 0.6)

5 Likely (0.6 - 0.8)

6 Very likely (0.8 - 1)

7 Certain (1)

You may only rank based on these discrete categories (1,2,3,4,5,6, or 7), no in-betweens are acceptable. Further, giving two or more Likert rankings to avoid definitively deciding is not allowed; when in doubt, you must provide your best single Likert ranking, and instead you may acknowledge any difficulties you had in coming to a final single Likert ranking within your follow-up rationale/explanation, but still you must provide a single discrete Likert ranking as your final answer, as this data will ultimately be entered categorically into an Excel file.

Please additionally provide a rationale/explanation behind why you decided to give the ranking you did.

Here is the specified statement which you are completing by filling in the end with your chosen Likert-ranked option for this trial: 

***“Insert Statement Here (via Coded Script)”***

***Category 6 Prompt:***

This is part of a study about what the future of AI in pathology will look like by 2030. The expert answers to Round 1 have been transformed into a series of statements, which will now be assessed by 1) humans and 2) AI models like yourself. In this round, you will be asked to rate each of the specified statements provided to you from the CSV file according to the Likert scale below. You will rate each specified statement one trial at a time, one specified statement per trial (i.e. one trial at a time, with each trial regarding only one specified statement).

Please answer considering only AI input, not digital pathology in a broad sense. Also, please

answer according to what you believe will happen by 2030, instead of what you would like to

happen.

All of your responses will remain anonymous to the rest of the panel experts.

Please estimate the PROBABILITY of this application of integrated diagnostics being used routinely by 2030, by selecting one of the following Likert scale responses below with which to complete the specified statement.

1 Impossible (0)

2 Very unlikely (0 - 0.2)

3 Unlikely (0.2 - 0.4)

4 Even chance/neutral (0.4 - 0.6)

5 Likely (0.6 - 0.8)

6 Very likely (0.8 - 1)

7 Certain (1)

You may only rank based on these discrete categories (1,2,3,4,5,6, or 7), no in-betweens are acceptable. Further, giving two or more Likert rankings to avoid definitively deciding is not allowed; when in doubt, you must provide your best single Likert ranking, and instead you may acknowledge any difficulties you had in coming to a final single Likert ranking within your follow-up rationale/explanation, but still you must provide a single discrete Likert ranking as your final answer, as this data will ultimately be entered categorically into an Excel file.

Please additionally provide a rationale/explanation behind why you decided to give the ranking you did.

Here is the specified statement which you are completing by filling in the end with your chosen Likert-ranked option for this trial:

***“Insert Statement Here (via Coded Script)”***

***Category 7 Prompt:***

This is part of a study about what the future of AI in pathology will look like by 2030. The expert answers to Round 1 have been transformed into a series of statements, which will now be assessed by 1) humans and 2) AI models like yourself. In this round, you will be asked to rate each of the specified statements provided to you from the CSV file according to the Likert scale below. You will rate each specified statement one trial at a time, one specified statement per trial (i.e. one trial at a time, with each trial regarding only one specified statement).

Please answer considering only AI input, not digital pathology in a broad sense. Also, please

answer according to what you believe will happen by 2030, instead of what you would like to

happen.

All of your responses will remain anonymous to the rest of the panel experts.

Please estimate the PROBABILITY that this task will become FULLY DELEGATED to AI and thus done in a FULLY AUTOMATED WAY in pathology labs by 2030, by selecting one of the following Likert scale responses with which to complete the specified statement:

1 Impossible (0)

2 Very unlikely (0 - 0.2)

3 Unlikely (0.2 - 0.4)

4 Even chance/neutral (0.4 - 0.6)

5 Likely (0.6 - 0.8)

6 Very likely (0.8 - 1)

7 Certain (1)

You may only rank based on these discrete categories (1,2,3,4,5,6, or 7), no in-betweens are acceptable. Further, giving two or more Likert rankings to avoid definitively deciding is not allowed; when in doubt, you must provide your best single Likert ranking, and instead you may acknowledge any difficulties you had in coming to a final single Likert ranking within your follow-up rationale/explanation, but still you must provide a single discrete Likert ranking as your final answer, as this data will ultimately be entered categorically into an Excel file.

Please additionally provide a rationale/explanation behind why you decided to give the ranking you did.

Here is the specified statement which you are completing by filling in the end with your chosen Likert-ranked option for this trial:

***“Insert Statement Here (via Coded Script)”***
